# Myomectomy in infertile women: More harm than good?

**DOI:** 10.3389/fsurg.2023.1151901

**Published:** 2023-04-17

**Authors:** Antonio Mercorio, Luigi Della Corte, Dominga Boccia, Mario Palumbo, Sabrina Reppuccia, Cira Buonfantino, Lara Cuomo, Maria Borgo, Antonio Zitiello, Maria Chiara De Angelis, Antonio Simone Laganà, Giuseppe Bifulco, Pierluigi Giampaolino

**Affiliations:** ^1^ Department of Public Health, University of Naples Federico II, Naples, Italy; ^2^ Department of Neuroscience, Reproductive Sciences and Dentistry, School of Medicine, University of Naples Federico II, Naples, Italy; ^3^ Department of Woman Mother Child, Lausanne University Hospital, Lausanne, Switzerland; ^4^ Unit of Gynecologic Oncology, ARNAS “Civico-Di Cristina-Benfratelli”, Department of Health Promotion, Mother and Child Care, Internal Medicine and Medical Specialties (PROMISE), University of Palermo, Palermo, Italy

**Keywords:** myomectomy, infertility, adhesion, laparoscopy, uterine fibroids

## Abstract

Adhesion formation following gynecological surgery remains a challenge. The adoption of minimally invasive surgical approaches, such as conventional or robotic-assisted laparoscopy combined with meticulous microsurgical principles and the application of adhesion–reducing substances, is able to reduce the risk of *de novo* adhesion formation but do not eliminate it entirely. Myomectomy is the most adhesiogenic surgical procedure and postoperative adhesions can have a significant impact on the ability to conceive. Therefore, when surgery is performed as infertility treatment, attention should be paid to whether the benefits outweigh the risks. Among several factors, the size and the location of fibroids are the most accountable factors in terms of adhesion development and post surgical infertility; therefore, the search for effective strategies against adhesion formation in this setting is of paramount importance. The purpose of this review is to evaluate the incidence and factors of adhesion formation and the best preventive measures current available.

## 1. Introduction

Uterine fibroids are the most common benign gynecologic disease in women of reproductive age. Fibroids can lead to a variety of symptoms including abnormal uterine bleeding, pain, pelvic heaviness, and can be the cause of infertility and obstetrical complications (1).

**Figure 1:**
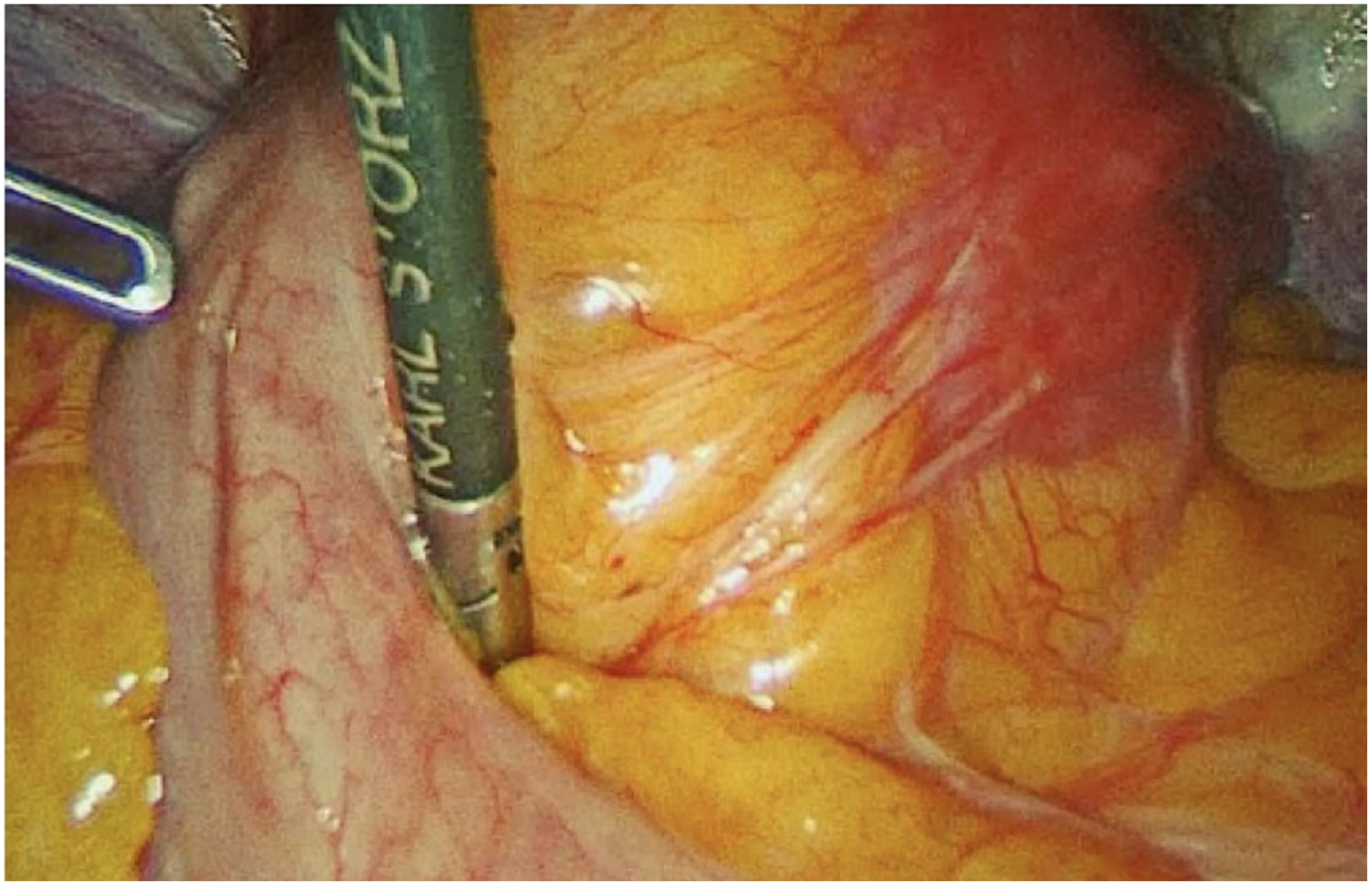
Adhesions through the posterior uterine wall and bowel. Sixteen months after laparoscopic myomectomy (Posterior uterine fibroid of 6 cm FIGO 4).

Many hypotheses have been proposed to explain how fibroids might cause infertility such as increased uterine contractility, deranged cytokine profile, abnormal vascularization, and chronic inflammation. However, a direct causal relationship between the presence of fibroids and infertility and the real benefit of myomectomy is yet to be defined (2). Moreover, It is beyond doubt that myomectomy in itself, being a major invasive procedure, runs the risk of damage to uterine myometrium, and endometrium as well as of producing scar tissue within the pelvic cavity (3). Postoperative adhesions are a well-known complication of myomectomy (4). Whether these adhesions really decrease the chance of getting pregnant remains an enigma; however, posterior wall myomectomy could have a special relevance considering the risk of adnexa involvement (Figure 1). Therefore, in women with otherwise unexplained infertility or requiring treatment for symptomatic fibroids, the surgeon must balance the benefit of such procedure in terms of fertility improvement on the one hand, and the consequences derived from adhesion development on the other, avoiding unnecessary myomectomies and involuntary iatrogenic damages (5). The purpose of this review is to help the surgeon in this difficult task, focusing on three main aspects: a) incidence and severity of adhesion after myomectomy; b) effectiveness of myomectomy in fertility enhancement; c) preventive measures available to minimize the risk and the consequences of adhesions development.

## 2. Methods

A literature search was performed on PubMed, Web of Science, Scopus and Cochrane Library using the search terms “myomectomy” alone and in combination with “adhesion”, “infertility outcome” and “medical treatment/management therapy”. No language restrictions were applied. Preferably, randomized controlled trials and systematic reviews including randomized controlled trials and/or cohort studies were included.

The latest search was performed on December 2022.

## 3. Adhesion post myomectomy

Post-operative pelvic adhesions have been reported to vary between 25% and 92% (6) and myomectomy is believed to be the most adhesiogenic surgical pelvic procedure (7). According to a panel of European experts (Anti-Adhesion in Gynaecology Expert Group—“ANGEL” and the European Society of Gynaecological Endoscopy—“ESGE”), all patients undergoing abdominal surgery should be informed about the risks and consequences of postoperative adhesions (8).

### 3.1. Abdominal vs. laparoscopic myomectomy

Several studies have investigated the occurrence of adhesions after laparoscopic myomectomy (LM) and abdominal myomectomy (AM). The reported rate of adhesion in AM has been estimated to vary between 28.1% (9) and 81% (10). A similar incidence has been reported after LM, ranging from 22.6% (9) to 88% (11). A randomized controlled trial (RCT) conducted by Tinelli et al. (9) provides a good comparison of adhesion development following AM and LM. These authors prospectively investigated the effect of an anti-adhesion agent (Interceed®) in a large cohort of patients (n = 546) with comparable baseline characteristics and found no difference in fibroid size; at a second-look laparoscopy, they found an incidence of adhesion development only slightly lower after LM compared to AM (28.1% vs. 22.6%) (9). Hence the risk of adhesion formation remains high even with LM, although it was hoped that laparoscopic myomectomy would minimize this. A possible explanation for this disappointing result might come from animal studies demonstrating that pneumoperitoneum itself can be an adhesiogenic factor (12). Hopefully, the recent introduction of insufflators, which deliver warmed and humidified gas, could help minimize post-surgical adhesion development (13).

To date, besides these aspects, laparoscopic myomectomy is preferred over open myomectomy due to its advantages in terms of postoperative pain, reduced risk of postoperative infection, and shorter hospital stay (14); it must be acknowledged that the size of the fibroid, especially if greater than10 cm, is a limiting factor for a mini invasive approach; in these cases, the difficulty and the time required for the specimen extraction, should be not underestimated (15).

In addition, the location and the number of fibroids can be other important limiting factors for a laparoscopic surgery.

Robotic surgery is an emerging modality, offering the possibility of performing fast and effective sutures in a short time and exploiting different angles. This surgical approach will be able to guarantee the patient the effectiveness of an open myomectomy with the advantages in terms of recovery of a minimally invasive technique (16).

### 3.2. Location and fibroid size as cofactor of adhesions development

The prevalence of adhesions varies according to fibroid location; in fact, reduction of fertility is minimal following fundal and anterior incisions compared to posterior incisions where the involvement of the adnexa within the scar commonly occurs. In one study, postoperative adhesions were found in 94% of patients with posterior wall incisions and only in 55% when the incision involved the anterior uterine wall (17). In the 90's, Keckstein et al. (7) and Dubuisson et al. (18), aiming to enable adhesion lysis after myomectomy and to assess the quality of myomectomy scars, suggested an early second-look laparoscopy (SLL) systematically after posterior laparoscopic myomectomy. In agreement with their recommendations, SLL was performed in multiple studies, providing interesting results: in addition to the location, fibroid size and incision length were found responsible cofactors of a higher incidence and severity of adhesion formation.

In their prospective multicenter study, Diamond et al. included one hundred twenty-seven women who underwent uterine myomectomy with at least one posterior uterine incision >1 cm in length, and found at least one adnexa totally free of adhesions in only 31% of patients (19). Coddington et al., in a study involving 20 patients who had an abdominal myomectomy followed by a SLL, observed that for every additional centimeter of incision length, the total adhesion area over the uterine serosal increased by 0.55 cm^2^ (20).

Trew et al. studied the impact of several surgical factors (blood loss, duration, number of incisions, number of knots) and found a significant association between incision length >5 cm and adhesion development (21). A correlation between incision length and adhesions was reported by Kumakiri et al.; in their study, patients presenting adhesions had a median total incision length of 10 cm (range, 4.6 cm–17.5 cm) whereas patients having a median incision length of 8 cm (range, 2 cm–23.9 cm) did not develop adhesion (22).

In another study, Takeuchi et al. aimed to determine the factors influencing the development of postoperative adhesions and found that fibroid diameter influenced the incidence of *de novo* adhesions (23). Accordingly, the enucleation of a large fibroid and the length of the incision did not lead to the formation of a smooth wound due to a redundant serosa, and the resulting wound protrusion is a critical factor influencing adhesion. Therefore, these authors recommend an accurate reconstruction of the uterine wall, trimming or burying the redundant tissue after the removal of fibroids to prevent adhesion development.

The results of the abovementioned studies lead us to conclude that myomectomy is a very adhesiogenic procedure and, more important, that posterior myomectomy is burdened by a high incidence of adhesion. In these cases, the potential involvement of the adnexa can result in post-surgical fertility impairment. Therefore, it is advisable, when deciding the management of this clinical condition, to weigh the real benefit of surgical therapy against the risk of unintentional post-surgical infertility. Unfortunately, as underlined very recently by Freytag et al. (24), studies addressing the question of the potential benefit of myomectomy are very far from providing definitive conclusions.

## 4. Does myomectomy improve fertility outcomes?

**Figure 2:**
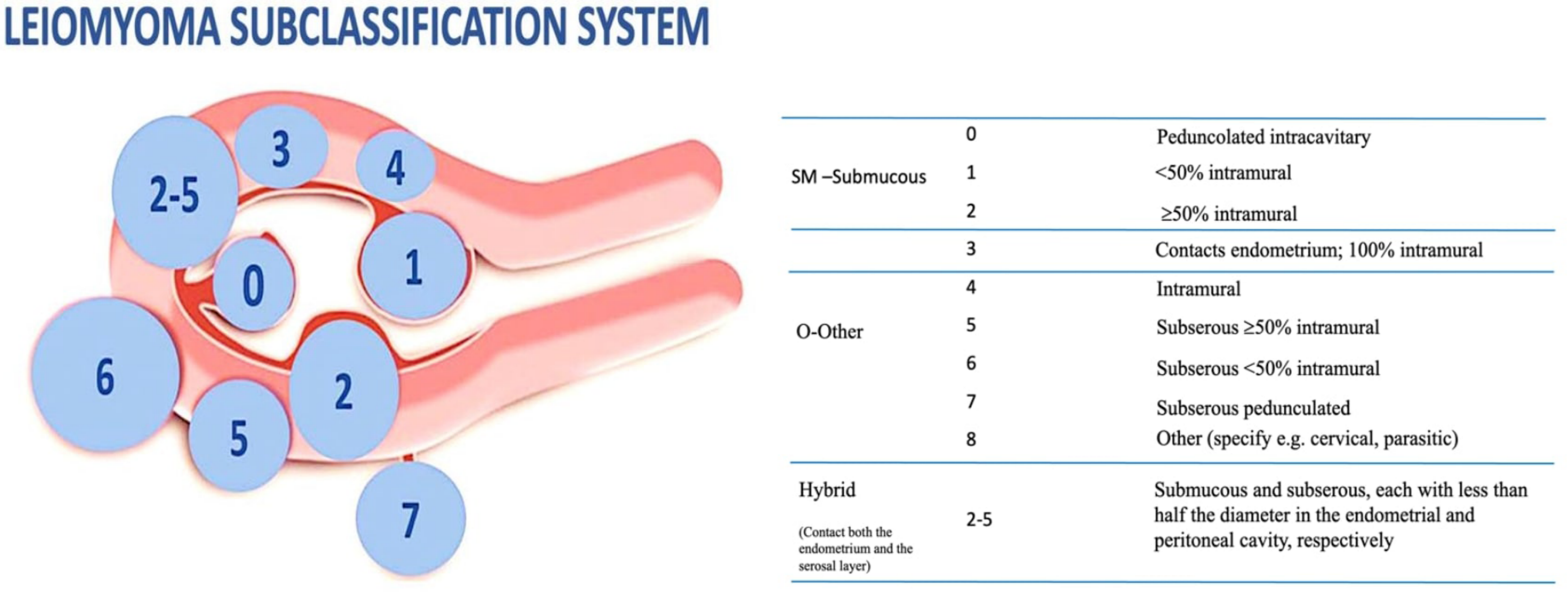
FIGO classification of uterine fibroids according to Munro et al. (2018).

The question whether myomectomy leads to improved fertility has been addressed by a recent review that examined the correlation between reproductive outcomes and locations of fibroids (25). While subserosal fibroids do not appear to affect fertility, fibroids distorting the uterine cavity are generally acknowledged to disturb implantation (26), and the need to treat them is widely accepted. On the other hand, the role in the genesis of infertility and the benefits of myomectomy on reproductive outcomes for intramural fibroid [type 3 to 5 according to the recent International Federation of Gynecology and Obstetrics (FIGO) classification] is less clear (27) (Figure 2).

### 4.1. The lesson learned from IVF

In women with fibroids, *in vitro* fertilization (IVF) is a model capable to elucidate the relationship between fibroids and infertility. Studies comparing the outcome of IVF cycles in women with intramural fibroid vs. women without fibroid seem to demonstrate a significant negative impact of intramural fibroids on fertility potential and recommend surgical removal of fibroids before IVF. Wang et al. conducted an updated systematic review of 28 studies involving 9,189 patients and reported, for intramural fibroids, a significant reduction of blastocyst implantation and live birth rates (28).

Rikhraj et al. reviewed 15 studies enrolling patients with non-cavity-distorting intramural fibroids undergoing IVF and found 44% lower odds of live birth and 32% lower odds of a clinical pregnancy compared to women without fibroids (29). Unfortunately, these reviews do not provide clear data on the size and location of the fibroids considered by Donnez et al. and Dolmans et al. as cofactors possibly accounting for the negative effects of fibroids on fertility (30).

### 4.2. On the relationship between location and size of fibroid

Through the analysis of many studies published, the above-mentioned authors conclude that the concurrent size and proximity to the uterine cavity of a fibroid are essential for unfolding the negative effect on fertility of intramural fibroids.

Accordingly, a fibroid of just 2 cm located close to the endometrial lining (type 3) will have a detrimental effect on fertility outcome; differently, in the case of intramural fibroid not in contact with the underlying endometrium (type 4, 5), 3 cm is the cut-off size considered to establish a fertility impairment (31).

The rationale for this statement lies in the fact that the negative impact of intramural fibroids can be mediated by signaling molecules produced by the fibroid able to reach the endometrial cavity, inducing an adverse effect on the homeostasis and receptivity of the endometrium (32).

However, a consensus regarding the size of a fibroid to be considered indicative of fertility impairment is still far from being reached.

For example, Yan et al. (33) noted a significant negative effect on delivery rate when women who underwent IVF with intramural fibroid with a diameter >3 cm, irrespective of location, were compared with a matched control group. These results were confirmed by the same authors in a large retrospective study including 151 cases and 453 matched controls (34), and by Christopoulos et al. (35) who found no difference in pregnancy outcome in women undergoing IVF with one fibroid <3 cm compared with controls.

On the other hand, Behbehani et al. examined a total of 929 fresh single-blastocyst transfer cycles and found that even a single and relatively small intramural fibroid (>1.5 cm) was able to affect clinical pregnancy and live birth rates (36); conversely, Somigliana et al. in a prospective study failed to observe a detrimental effect on IVF outcome in the presence of fibroids smaller than 5 cm and not distorting the endometrial contour (37).

As reviewed, these studies do not provide definitive conclusions on the relationship between intramural fibroids and fertility impairment. If we add to this uncertainty the risks of impaired fertility due to post-surgical adhesion development, especially in case of posterior fibroid, it is evident that the surgical option must be carefully evaluated, adopting all the measures to minimize adhesion development.

## 5. Adhesion prevention

For women wishing to conceive, effective adhesion prevention after myomectomy is essential, which requires appropriate surgical techniques (38). Gentler handling and precise dissection of anatomical structures are mandatory and can be easily achieved thanks to the magnified view provided by laparoscopy (39). The larger the residual amount of blood, the more frequently adhesions can occur, therefore it is essential that complete hemostasis is achieved, paying attention however to reduce cautery time and aspirate aerosolized tissue following this procedure.

Frequent irrigation of the abdominal cavity during and at the end of surgery with a large amount of Ringer's lactate should be followed (40) (Table 1).

**Table 1:** Strategy for adhesion reduction during myomectomy.

Adhesion-reduction steps during myomectomy
Perform diligent hemostasis but ensure diligent use of cautery
Reduce risk of infection
Limit use of sutures and choose fine nonreactive sutures
Reduce duration of surgery
Avoid foreign bodies—such as materials with loose fibers
Reduce drying of tissues (limit heat and light)
**In laparotomic surgery**
Minimal use of dry towels or sponges
Use starch- and latex-free gloves
**In laparoscopic surgery**
Use frequent irrigation and aspiration
Reduce pressure and duration of pneumoperitoneum

In the literature, there is a lack of agreement regarding the type of suture and the technique that should be adopted in order to prevent adhesion formation. A higher number of knots, however, seems to be associated with a higher adhesion rate (21). Therefore, to prevent adhesions development, a running suture should be preferred to single stitches. Regarding the choice of suture material, it must be considered that monofilament tends to be less reactive and cause less of an inflammatory response compared to multifilament; however, due to its greater memory and lower coefficient of friction, it is not always the preferred choice by the surgeon.

Finally, the barbed suture that does not require the tying of knots and has been proven to facilitate laparoscopic myomectomy by reducing the total operative time, seems to have a similar impact on reproductive outcomes as smooth conventional threads (41).

Disappointingly, these measures have not proven to be sufficient, and even the results of antiadhesion agents are considered only partially satisfactory (42, 43): the most frequently utilized products are physical barriers used to prevent adhesion formation (e.g., INTERCEED, Ethicon, Somerville, NJ, United States; SEPRAFILM,Baxter, Deerfield, IL, United States.) Physical barriers do not interact with the process of adhesion formation but only act as a spacer separating the surfaces of the wound surfaces during the first phase of tissue regeneration.

New therapies able to affect the underlying pathophysiology of adhesion formation will provide new opportunities to treat this complication (44). Although there is insufficient evidence to support the routinary adoption of these recommendation in every myomectomy, in case of posterior myomectomy they should nevertheless be particularly recommended, taking into account the high rate of post-surgical adhesions involving adnexa.

### 5.1. Fibroid shrinkage

A very important predictor of adhesion formation after myomectomy is the length of the incision into the uterine surface.

Methods to keep incision length to a minimum through the preoperative reduction of fibroid size seem to be sound surgical judgment. Currently, GnRH agonists (GnRH-a) and selective progesterone receptor modulators (SPRMs) are the medical therapies with the best evidence of fibroid volume reduction. Short-term pre-operative treatment with GnRH-a may decrease the risk of post-operative adhesion through significant fibroid size reduction (45).

A systematic review of 26 randomized controlled trials confirmed the therapeutic benefits of GnRH-a before myomectomy (46). A reduction in fibroid size up to 55.6% has been reported in a recent study with subcutaneous injections of goserelin 3.75 mg administered twice before surgery at 4-week intervals (47). A crucial question so far unanswered is the effectiveness of shrinkage in the prevention or reduction of adhesion development. Unfortunately, the only prospective randomized study available is the one by Coddington et al. where some doubts have been raised on the effectiveness of this therapy (20). This trial included 20 patients assigned randomly to receive either GnRH -a or placebo three months before the initial surgery, followed by second-look laparoscopy two to ten weeks later to evaluate postoperative adhesions; the authors found that presurgical GnRH-a treatment did not decrease adhesion formation compared with placebo. However, apart for the small sized sample, a great limitation of this study is a lack of detailed data on pre- and post-treatment size of the fibroids, which does not allow definitive conclusions to be reached on the role of GnRH-a as preventive measure of adhesion development.

An additional positive effect of GnRH-a on adhesion development can be ascribed to the modification in the coagulation and fibrinolytic system in response to the induced hypoestrogenism (48).

Inflammation plays a pivotal role in adhesion development, and the hypoestrogenic milieu produces an anti-inflammatory effect through the reduction of estrogen dependent inflammatory factors (angiogenic growth factors, epidermal growth factors, and platelet derived growth factor); to this regard, experimental studies on the rodent model with uterine serosal injury have confirmed this positive effect induced by GnRHa in adhesion prevention (49).

Alternative pharmacological agents for the pre-surgical treatment of fibroid have been evaluated more recently with the development of selective progesterone receptor modulators (SPRMs) and GnRH antagonist.

GnRH antagonists, acting immediately to suppress the secretion of FSH and LH by blocking pituitary GnRH receptors, were significantly more effective than placebo in decreasing uterine fibroid size (50, 51). An open-label study based on 19 patients reported that ganirelix was able to decrease fibroid and total uterine volumes as early as 19 days after initiation of treatment (52). The growing evidence of the crucial role of progesterone in the pathophysiology of uterine fibroids has promoted clinical studies on the role of the SPRMs for the preoperative treatment of uterine fibroids (53). Ulipristal acetate (UPA) is a SPRM without hypoestrogenic effects, previously approved for the pre-operative treatment of symptomatic fibroids (54) and, at present temporarily withdrawn from the market because of safety concerns linked to some cases of liver injury (55). The difference in terms of efficacy between UPA and GnRH-antagonist has not yet been clearly defined. If, on one hand, a double-blind randomized controlled trial demonstrated that GnRH-a pretreatment was associated with a greater reduction in volume than UPA (−47% with GnRH antagonist compared to −20% with 5 mg UPA for up to 13 weeks treatment) (53), on the other hand, a randomized trial by Donnez et al. – comparing UPA with GnRH-a, failed to show significant differences in fibroid volume reduction after 3 months of pretreatment between the two groups (54).

### 5.2. Second look laparoscopy

Second-look laparoscopy (SLL) is a feasible procedure performed within a certain lapse of time after the initial operation to diagnose and treat all newly-formed pelvic adhesion even if burdened by the risk of adhesions reformation limiting at some extents the efficacy of these procedure (56). To date, scanty data on the reproductive outcome of patients after SLL has been published.

A recent systematic review based on 5 randomized controlled trials has failed to show significant benefits on fertility outcome following SLL adhesiolysis; however, as reported by the authors, this conclusion was based on studies either of poor quality or underpowered (57).

Kubinova et al. (58). specifically addressed the reproductive outcome after laparoscopic/laparotomic myomectomy by comparing patients who underwent SLL procedure (including adhesiolysis) with a group of patients with no SLL intervention. Even though the occurrence of adnexal adhesions in patients after open myomectomy undergoing SLL procedure was higher, they have found no statistical difference in pregnancy rates compared with the no intervention group. Therefore the effect on fertility of adhesiolysis remain questionable.

However, interesting data come from a recent clinical trial by Li et al. on a large number (*n *= 216) of women who initially underwent laparoscopic salpingostomy for ectopic pregnancy, followed by randomization at 3 months to SLL and adhesiolysis or no intervention (59).

In this study, the overall pregnancy rate did not differ between the two groups; however, stratifying the patients further, comparing women who had only slight adhesions with those presenting severe adhesions at their first surgery, the improved subsequent fertility outcomes after SLL and adhesiolysis was more significant in the presence of severe and extensive adhesions.

Considering these results, Frishman G. N. (60) in an Editorial on “The Journal of Minimally invasive Gynecology” recommends as study methodology to establish the effect of SLL on reproductive outcome—if any—the sole inclusion of women with severe adhesion, considering them the best candidates to benefit from this procedure.

In the editorial, Frishman states that “consideration must be given to study SSL only in women undergoing myomectomy who required a posterior incision” (60).

A further consideration is reserved to the optimal time for performing SLL, which still remain matter of debate. It is generally believed that adhesion formation occurs in the first 3 to 5 days following surgery. In fact, some authors recommend very early SLL (within 7 days) (61); others believe that the early fine fibrinous adhesions are a normal consequence of tissue repair, due to local release of breakdown mediators in the remodeling process, and will eventually disappear with time and therefore recommend SLL between the time of serosal healing (eight days) and when fibrotic adhesion can be considered permanent (21 days) (62).

Finally, and not to be underestimated, is the benefit of SLL in planning of future fertility treatment, for example favoring the referral for IVF/ICSI for women deemed unlikely to conceive based on post-surgical laparoscopic appearance of the pelvis. The advent of mini-laparoscopy and the use of regional anesthesia for laparoscopic surgery, with rapid recovery time following day surgery, can encourage (in selected cases) this procedure (63).

### 5.3. Ovariopexy

The basic concept of transient ovariopexy arises from the purpose to keep the ovary away from the injured peritoneum whenever it is desirable to prevent the ovary from being concealed in the fibrous adhesive band involving the pouch of Douglas, negatively affecting the reproductive potential. This procedure has been for years described as a safe, simple, and excellent tool in the prevention of post-operative ovarian adhesion in women undergoing surgery for endometriosis (64). The technique involves a loose, temporary suspension of the ovary trough the use of a synthetic absorbable/ nonabsorbable monofilament suture to the anterolateral abdominal wall or, less frequently, to the round ipsilateral ligament (65). The lapse of time after which the suspended ovaries should be released from the abdominal wall is debatable and varies between 5 days (66) and 7–9 days, as suggested by Trehan et al. (67), to allow a complete absorption of the blood in the cavity—which is a major risk for adhesion formation. In addition, ovariopexy may be of benefit for patients who develop severe adhesion after posterior myomectomy, because it may facilitate the subsequent IVF/ICSI, which requires appropriate pelvic positioning of the ovary for successful oocyte retrieval.

## 6. Conclusion

When the workup for infertility reveals a fibroid, the efficiency of myomectomy in restoring fertility must be adequately weighed against the risks of adhesion development, with proper selection of the patients. This rigorous approach is particularly relevant in case of posterior-located large fibroids due to the high risk of adnexal adhesion formation that may adversely affect reproductive function. It is therefore essential to determine when myomectomy is to be considered beneficial and, if so, adopt all the measures available to avoid post surgical infertility when the initial procedure is performed for fertility enhancement. Many emerging alternative techniques will likely further decrease surgical myomectomies especially when fertility preservation is the goal. However, many of these have not been employed on a large scale, and data on the reproductive outcomes for patients trying to conceive are insufficient to make recommendations.
